# Direct and Indirect Inhibition of *Salmonella* Peptide Deformylase by Nitric Oxide

**DOI:** 10.1128/mBio.01383-20

**Published:** 2020-11-17

**Authors:** Anshika Singhal, Ferric C. Fang

**Affiliations:** a Department of Laboratory Medicine and Pathology, University of Washington, Seattle, Washington, USA; b Department of Microbiology, University of Washington, Seattle, Washington, USA; University of Illinois at Chicago

**Keywords:** mismetallation, peptide deformylase, salmonella, nitric oxide, zinc homeostasis

## Abstract

Salmonella enterica serovar Typhimurium is an intracellular pathogen that elicits nitric oxide (NO·) production by host macrophages. NO· is a potent antimicrobial mediator with diverse targets, including protein thiols and metal centers. The mobilization of zinc from metalloproteins by NO· increases the availability of free intracellular zinc, which is detrimental to bacterial cells, but the precise mechanism of zinc cytotoxicity is uncertain. Here, we show that excess zinc results in the mismetallation of the essential iron-containing enzyme peptide deformylase (PDF), thereby diminishing its activity. PDF mismetallation is observed in zinc-treated bacteria lacking the zinc exporters ZntA and ZitB and is also observed during nitrosative stress, suggesting that NO·-mediated zinc mobilization results in PDF mismetallation. However, NO· also inhibits PDF directly by *S-*nitrosylating the metal-binding Cys90 residue. These observations identify PDF as an essential bacterial protein that is subject to both direct and indirect inactivation by NO·, providing a novel mechanism of zinc toxicity and NO·-mediated antibacterial activity.

## Abstract (precis)

We have previously shown that the host-derived antimicrobial mediator nitric oxide (NO·) mobilizes zinc from bacterial metalloproteins. The present study demonstrates that NO· inactivates the essential iron-containing enzyme peptide deformylase, both by promoting its mismetallation by zinc and by directly modifying its metal-binding site. We explain how free intracellular zinc is detrimental for cells and reveal a new mechanism of NO·-mediated bacterial growth inhibition that is distinct from previously known targets.

## INTRODUCTION

Nitric oxide (NO·) is an antimicrobial mediator generated by the inducible nitric oxide synthase of phagocytic cells in response to infection (1). NO· primarily inhibits microbes by targeting thiols and metal centers of proteins required for DNA replication and diverse metabolic processes (2–6). The *S-*nitrosylation of cysteine ligands can mobilize free iron (7) and zinc from metalloproteins (4, 8). NO· has been shown to mobilize zinc by targeting cysteine-containing proteins in Escherichia coli and Borrelia burgdorferi (9, 10). Zinc is an essential metal in living cells and a structural or catalytic component of hundreds of enzymes (11). In Salmonella enterica serovar Typhimurium, zinc metalloproteins constitute nearly 10% of all the protein targets of NO·-mediated *S-*nitrosylation (8). When NO·-producing macrophages internalize *S.* Typhimurium, mobilized free intracellular zinc can be detected in mutant *Salmonella* strains that are deficient in zinc efflux (8). NO· treatment leads to zinc mobilization and the arrest of cell division in *S*. Typhimurium due to the *S*-nitrosylation of enzymes involved in DNA replication and repair (4, 8).

Protein metallation is regulated in several ways to ensure that the appropriate metal is bound by a metalloprotein. The relative affinities of metalloproteins for binding divalent metal ions follow the Irving-Williams series (12). Transition metal toxicity is prevented by regulated mechanisms of metal uptake, sequestration, and efflux (8, 13, 14). To prevent metals with a high binding affinity such as zinc from inappropriately binding to sites intended for more weakly binding metals, specific metallochaperones may be employed, and metal availability may be regulated both spatially and temporally (14, 15). As zinc is one of the most highly competitive metals in the Irving-Williams series (16), free zinc can have toxic effects resulting from the irreversible mismetallation of metalloenzymes that normally bind to other metals (17, 18).

Peptide deformylase (PDF) is a mononuclear iron-binding protein that removes the *N*-formyl methionine group from nascent polypeptides, which is an essential step in bacterial protein maturation (19). The deformylation of methionine is a prerequisite for the removal of the N-terminal methionine group, which occurs in more than half of all bacterial proteins (20). PDF is therefore essential for bacterial growth and survival and is viewed as an attractive target for antibacterial drug development (21). PDF was originally believed to be a zinc metallopeptidase but was subsequently shown to be an iron-binding protein with labile metal binding in aerobic environments (22, 23). The initial mischaracterization of PDF as a zinc metalloprotein was due to the presence of a characteristic HEXXH motif as well as the presence of bound zinc in purified protein preparations. Kinetic measurements using purified PDF protein reconstituted with either iron or zinc revealed that although zinc binds to PDF more tightly with an increased half-life of metal dissociation, it confers a substantial reduction of the catalytic rate constant (24).

The high affinity of PDF for zinc suggests that PDF may be a target of zinc mismetallation under stress conditions. We therefore hypothesized that zinc might replace iron in PDF under conditions of zinc excess or nitrosative stress. In the present study, we demonstrate that *Salmonella* PDF can be mismetallated under conditions of either zinc or nitrosative stress and that NO· can also directly inhibit PDF by *S*-nitrosylation.

## RESULTS

### PDF overexpression confers resistance to zinc stress.

**FIG 1:**
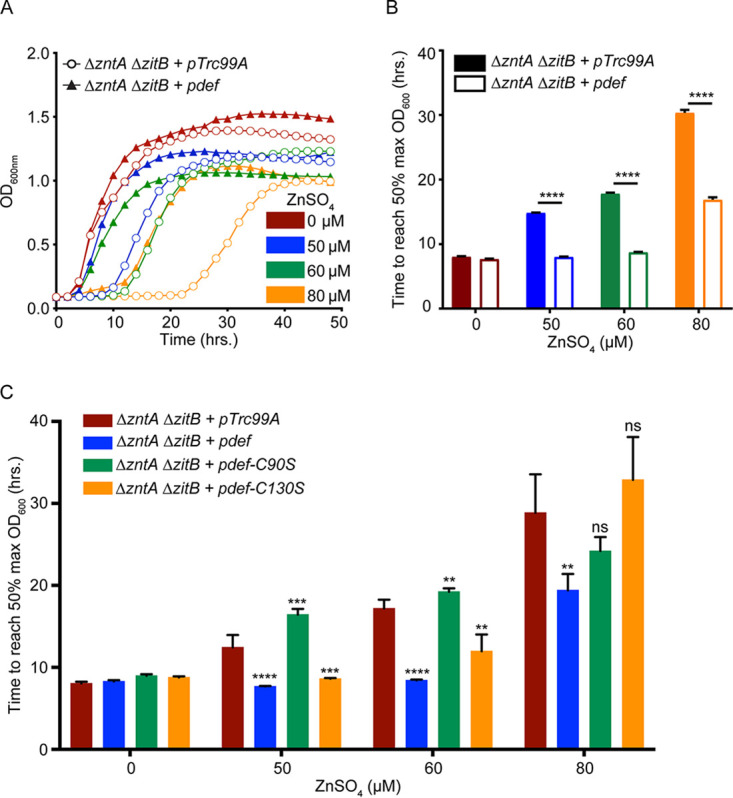
PDF overexpression restores growth in the presence of excess zinc. (A) A Δ*zntA* Δ*zitB* efflux-deficient mutant *S*. Typhimurium strain exhibits dose-dependent inhibition of exit from lag phase in the presence of supplemental ZnSO_4_. The overexpression of PDF from an inducible plasmid (p*def*) restores bacterial growth in the presence of zinc, in contrast to a vector control (pTrc99A). (B) The mean times required to reach the 50% maximum final OD_600_ in panel A were compared at different ZnSO_4_ concentrations in the absence or presence of PDF overexpression. (C) Overexpression of a PDF-C90S mutant (*pdef-C90S*) fails to restore the growth of the Δ*zntA* Δ*zitB* strain in the presence of ZnSO_4_, while overexpression of a PDF-C130S mutant (*pdef-C130S*) partially relieves zinc inhibition. Significance was determined by an unpaired two-tailed *t* test relative to the vector control. **, *P* ≤ 0.01; ***, *P* ≤ 0.001; ****, *P* ≤ 0.0001; ns, nonsignificant (*n* = 3 to 6). Data are represented as means, with error bars showing standard deviations.

*S.* Typhimurium has three known zinc efflux systems, of which ZntA and ZitB play a primary role (8). Bacterial strains deficient in both ZntA and ZitB exhibit a delayed exit from lag phase in the presence of excess zinc. We used a strain lacking ZntA and ZitB to prevent the rapid buffering of intracellular zinc levels and analyzed the metallation of PDF under these conditions. The gene encoding PDF (*def*) was placed under the control of an inducible promoter on a multicopy plasmid (p*def*), and growth was measured in the presence of excess zinc. A Δ*zntA* Δ*zitB* mutant carrying the plasmid vector exhibited a pronounced growth defect in the presence of excess zinc, but the p*def* plasmid restored growth in zinc-supplemented medium (Fig. 1A and B).

PDF contains an HEXXH metal-binding motif (25). In addition to the two histidine residues contained in this motif, a cysteine residue (Cys90) is also involved in metal binding and required for enzyme activity (see Fig. S1A in the supplemental material). A second cysteine residue (Cys130) is not involved in metal binding, but its replacement with serine results in an ∼50% reduction in enzyme activity (Fig. S1A). The overexpression of PDF-C90S failed to improve the growth of a Δ*zntA* Δ*zitB* mutant strain in the presence of excess zinc, whereas the overexpression of PDF-C130S only partially restored growth (Fig. 1C and Fig. S1B), even though it retains zinc binding. These observations suggest that PDF overexpression relieves zinc inhibition by restoring enzyme activity rather than by sequestering zinc.

10.1128/mBio.01383-20.1 FIG S1 PDF-WT but not PDF-C90S overexpression restores growth in the presence of excess zinc. (A) Comparison of enzyme activities of PDF-WT (blue), PDF-C90S (green), and PDF-C130S (orange) proteins in extracts prepared from WT *S.* Typhimurium. Activity is plotted as a percentage of the total activity relative to that of PDF-WT. Significance was determined by an unpaired two-tailed *t* test relative to PDF-WT. ***, *P* ≤ 0.001; ****, *P* ≤ 0.0001 (*n* = 3 to 6). Data are represented as means, with error bars showing standard deviations. (B) PDF-WT, PDF-C90S, and PDF-C130S proteins were overexpressed in an *S.* Typhimurium Δ*zntA* Δ*zitB* strain, and growth was measured in the presence of various concentrations of ZnSO_4_. Download FIG S1, TIF file, 1.1 MB. Copyright © 2020 Singhal and Fang. 2020 Singhal and Fang. This content is distributed under the terms of the Creative Commons Attribution 4.0 International license.

### Zinc mismetallation reduces PDF activity.

As PDF overexpression protects cells from zinc stress, we analyzed the effect of zinc binding on PDF enzymatic activity. Recombinant PDF was expressed in E. coli, purified, and chelated to remove all bound metals (apoPDF), followed by incubation with either iron or zinc before the measurement of PDF activity. Apoprotein lost all PDF activity, which could be restored by the subsequent addition of iron but not zinc. Zinc-bound PDF had approximately 10% activity compared with iron-bound PDF (Fig. S2A).

10.1128/mBio.01383-20.2 FIG S2 Zinc binding reduces PDF enzyme activity. (A) Purified GST-tagged PDF was treated with EDTA to remove the bound metal ion (apoPDF). The apoprotein was then incubated with excess quantities of Fe ammonium sulfate (iron) or Zn sulfate (zinc) and assayed for enzyme activity. (B) There is no statistically significant difference between the activities of PDF obtained from the WT and Δ*zntA* Δ*zitB* strains. Significance was determined by an unpaired two-tailed *t* test. ns, nonsignificant (*n* = 6). Data are represented as means, with error bars showing standard deviations. Download FIG S2, TIF file, 0.6 MB. Copyright © 2020 Singhal and Fang. 2020 Singhal and Fang. This content is distributed under the terms of the Creative Commons Attribution 4.0 International license.

**FIG 2:**
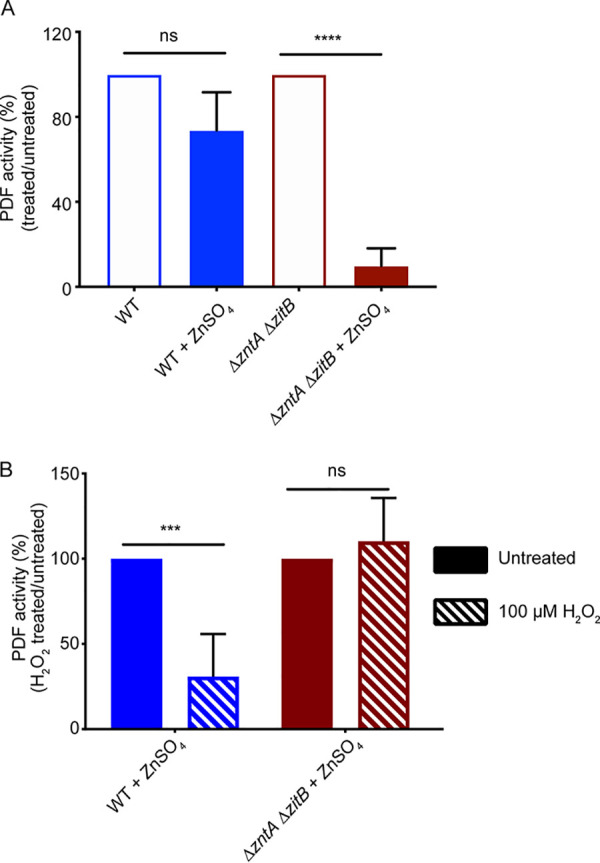
PDF is mismetallated under zinc stress. (A) ZnSO_4_ supplementation (125 μM) did not significantly impair PDF activity from WT extracts (filled blue bar) compared to untreated cells (open blue bar) but resulted in an ∼90% reduction in PDF activity in extracts from the efflux-deficient Δ*zntA* Δ*zitB* mutant (red bars). (B) Zinc-treated extracts (filled bars from panel A) were further treated with 100 μM H_2_O_2_ for 10 min before measurement of PDF activity. Activity is plotted as a percentage of the total activity of the respective H_2_O_2_-untreated extracts. Native iron-bound PDF is H_2_O_2_-sensitive, whereas the zinc-bound protein is H_2_O_2_-resistant. Residual enzyme activity in Δ*zntA* Δ*zitB* extracts was insensitive to H_2_O_2_ treatment (red bars), suggesting the presence of zinc-bound protein. Significance was determined by an unpaired two-tailed *t* test. ***, *P* ≤ 0.001; ****, *P* ≤ 0.0001; ns, nonsignificant (*n* = 3 [A] or 5 [B]). Data are represented as means, with error bars showing standard deviations.

Next, the effect of zinc supplementation on PDF enzyme activity was measured in *Salmonella in vivo*. Cells were grown in zinc-supplemented medium, and whole-cell protein extracts were used to measure PDF activity. The absolute activities of PDF in wild-type (WT) and Δ*zntA* Δ*zitB* strains in the absence of any stress were comparable (Fig. S2B). The activity of PDF in the WT extract was not significantly affected by zinc supplementation (Fig. 2A, blue bars). However, the PDF activity in extracts from a Δ*zntA* Δ*zitB* mutant was significantly lower than that in the same strain grown without zinc supplementation (Fig. 2A, red bars), suggesting that zinc mismetallation during zinc overload inhibits PDF activity.

To confirm that PDF from a Δ*zntA* Δ*zitB* mutant strain grown in zinc-supplemented medium was bound to zinc, we assessed the sensitivity of the enzyme to oxidative damage. Iron-bound PDF is highly sensitive to hydrogen peroxide (H_2_O_2_) due to the oxidation of ferrous ion and dissociation of the metal, whereas zinc-bound PDF is resistant to oxidative stress (23). Therefore, we incubated purified zinc-treated whole-cell protein extracts (Fig. 2A, filled bars) with H_2_O_2_ and then measured PDF activity. PDF from zinc-treated WT cells was highly sensitive to H_2_O_2_, indicating the presence of iron-bound PDF, whereas PDF from a zinc-treated Δ*zntA* Δ*zitB* mutant strain was H_2_O_2_ resistant (Fig. 2B). Together, these observations suggest that PDF is susceptible to zinc mismetallation with a loss of activity under zinc stress.

### NO· mobilizes intracellular zinc and inhibits PDF.

**FIG 3:**
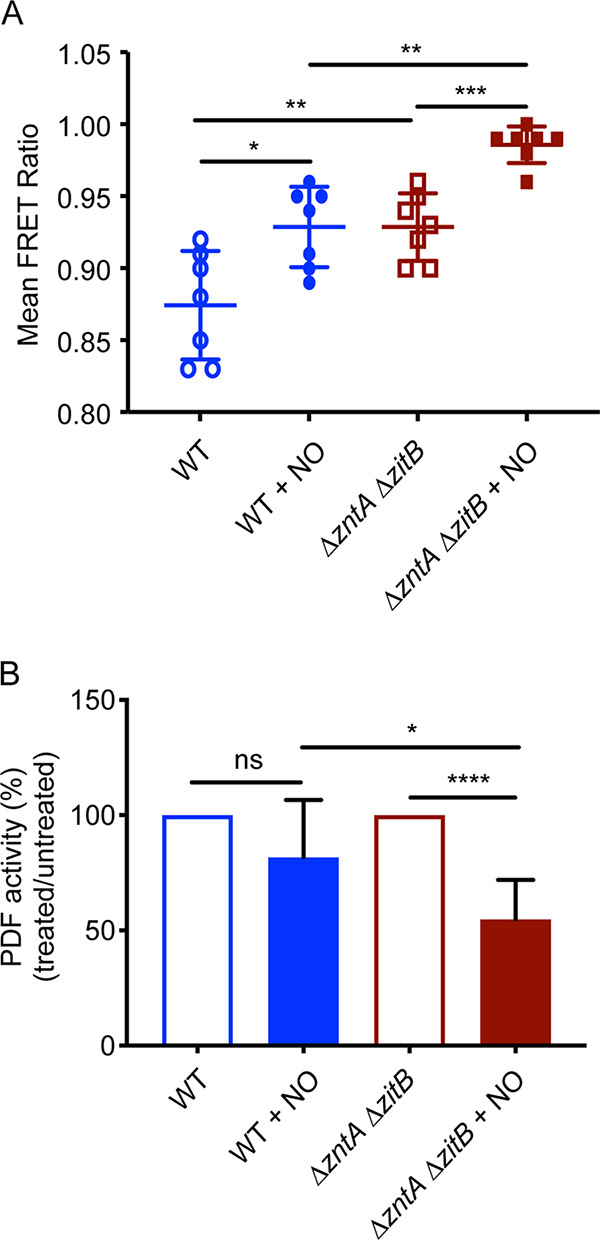
NO· causes zinc overload and PDF mismetallation. (A) Free intracellular zinc was measured using a genetically encoded biosensor. WT (blue) or Δ*zntA* Δ*zitB* (red) cells were treated with 500 μM spermine NO· for 1 h before measurement of zinc-dependent FRET. Both strains showed an increase in FRET, indicating zinc mobilization by NO·, with higher levels in the Δ*zntA* Δ*zitB* efflux-deficient mutant. (B) Peptide deformylase activity in WT extracts (blue) was not significantly different after treatment with 50 μM of the NO· donor spermine NONOate (SperNO) for 1 h, whereas PDF in Δ*zntA* Δ*zitB* extracts (red) exhibited an ∼50% loss of activity after NO· treatment. Significance was determined by an unpaired two-tailed *t* test. *, *P* ≤ 0.05; **, *P* ≤ 0.01; ***, *P* ≤ 0.001; ****, *P* ≤ 0.0001; ns, nonsignificant (*n* = 7). Data are represented as means, with error bars showing standard deviations.

Zinc efflux enhances *Salmonella* resistance to nitrosative stress (8), suggesting that nitric oxide (NO·) increases intracellular zinc levels. We measured intracellular free zinc levels in *S.* Typhimurium after treatment with NO· using a genetically encoded fluorescence-based zinc biosensor, ZapCV5 (22). This biosensor contains the first two zinc fingers of the Zap1 transcription factor from Saccharomyces cerevisiae, sandwiched between two fluorescent proteins, enhanced cyan fluorescent protein (CFP) and circularly permuted Venus (cp173Venus) (26). Zinc binding to the zinc finger domain results in fluorescence resonance energy transfer (FRET) from CFP to cpVenus. The biosensor was expressed from a plasmid and introduced into *S.* Typhimurium WT and Δ*zntA* Δ*zitB* strains. Cells were treated with the NO· donor spermine NONOate (SperNO), and the FRET response was measured. An increase in the FRET signal, indicative of free intracellular zinc, was observed in both WT and Δ*zntA* Δ*zitB* cells treated with the NO· donor but the signal was elevated in the zinc efflux-deficient strain even in the absence of nitrosative stress (Fig. 3A). The differences in the FRET ratios of unstressed WT and Δ*zntA* Δ*zitB* cells were not observed in a defined minimal medium, in contrast to Luria-Bertani (LB) medium, which contains larger amounts of zinc (Fig. S3A).

10.1128/mBio.01383-20.3 FIG S3 PDF in Δ*zntA* Δ*zitB* cells is more susceptible to NO· inhibition. (A) Free intracellular zinc was measured using a genetically-encoded biosensor. WT (blue) or Δ*zntA* Δ*zitB* (red) cells were left untreated in minimal growth medium for 1 h before measurement of zinc-dependent FRET. Both strains showed comparable FRET ratios. (B) PDF activity from WT extracts (blue) is not significantly different with 50 μM SperNO but decreases to ∼55% after treatment with 75 μM SperNO, whereas PDF activity in Δ*zntA* Δ*zitB* extracts (red) is significantly reduced after treatment with 50 μM SperNO. Significance was determined by an unpaired two-tailed *t* test. **, *P* ≤ 0.01; ***, *P* ≤ 0.001; ns, nonsignificant (*n* = 3 [A] or 4 [B]). Data are represented as means, with error bars showing standard deviations. Download FIG S3, TIF file, 0.8 MB. Copyright © 2020 Singhal and Fang. 2020 Singhal and Fang. This content is distributed under the terms of the Creative Commons Attribution 4.0 International license.

After establishing that NO· increases free zinc levels, we measured PDF activity to determine whether NO·-induced zinc mobilization correlates with a reduction in PDF activity. WT and Δ*zntA* Δ*zitB* strains containing p*def* were treated with 50 μM of the NO· donor SperNO, and PDF activity was measured in cell extracts. NO·-treated WT bacteria exhibited PDF activity comparable to that of untreated cells. However, NO·-treated Δ*zntA* Δ*zitB* mutant extracts contained significantly lower PDF activity than extracts from untreated cells (Fig. 3B). The higher levels of free zinc in NO·-treated Δ*zntA* Δ*zitB* cells (Fig. 3A) correlated with decreased PDF activity, consistent with zinc-dependent mismetallation of PDF during nitrosative stress under conditions in which elevated intracellular zinc concentrations cannot be ameliorated by zinc efflux. Higher concentrations of SperNO (75 μM) were able to reduce PDF activity in WT cells as well as in Δ*zntA* Δ*zitB* mutant cells (Fig. S3B).

### Zinc-independent inhibition of *Salmonella* by NO· in the presence of PDF.

**FIG 4:**
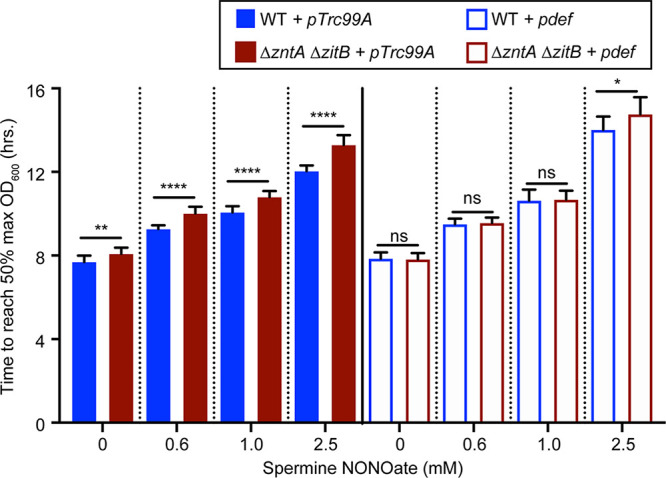
PDF overexpression abrogates WT and Δ*zntA* Δ*zitB* mutant differences but fails to restore growth in the presence of NO·. The mean time required to reach the 50% maximum final OD_600_ was measured for WT and Δ*zntA* Δ*zitB* mutant strains in the presence of various concentrations of the NO· donor SperNO. A Δ*zntA* Δ*zitB* mutant exhibited delayed exit from lag phase compared to the WT, which was abrogated by PDF overexpression (p*def*). However, dose-dependent NO· inhibition of the growth of both strains was still observed. Significance was determined by an unpaired two-tailed *t* test. *, *P* ≤ 0.05; **, *P* ≤ 0.01; ****, *P* ≤ 0.0001; ns, nonsignificant (*n* = 9 to 15). Data are represented as means, with error bars showing standard deviations.

To further verify the observations shown in Fig. 3, we compared the growth patterns of WT and Δ*zntA* Δ*zitB S*. Typhimurium strains during nitrosative stress. The growth of WT and Δ*zntA* Δ*zitB* mutant strains carrying p*def* was monitored in the presence of various concentrations of SperNO. The time required to reach the half-maximal optical density at 600 nm (OD_600_) was plotted as a measure of growth inhibition. In the absence of PDF overexpression, a Δ*zntA* Δ*zitB* mutant exhibited a delayed exit from lag phase compared with the WT in the presence of NO· (Fig. 4, filled bars). However, the overexpression of PDF restored the growth of the Δ*zntA* Δ*zitB* strain relative to the WT (Fig. 4, hollow bars), suggesting that zinc-dependent PDF inhibition is responsible for the enhanced sensitivity of a Δ*zntA* Δ*zitB* mutant to NO·. PDF overexpression did not completely restore growth to untreated levels in both WT and Δ*zntA* Δ*zitB* strains, presumably because NO· targets multiple other thiol and metal centers in *Salmonella*, which also contribute to growth inhibition.

Although PDF overexpression restored *Salmonella* growth during zinc overload (Fig. 1), p*def* failed to restore normal growth rates of the WT or the Δ*zntA* Δ*zitB* strain treated with NO· and actually exacerbated growth inhibition relative to a vector control (Fig. S4), suggesting that NO· might also inhibit PDF by a zinc-independent mechanism.

10.1128/mBio.01383-20.4 FIG S4 PDF overexpression fails to restore growth in the presence of NO·. The data from Fig. 4 are replotted to visualize different comparisons. PDF-overexpressing derivatives of both WT and Δ*zntA* Δ*zitB* strains are more sensitive to inhibition by NO· than the vector control at higher concentrations of SperNO. Significance was determined by an unpaired two-tailed *t* test. *, *P* ≤ 0.05; **, *P* ≤ 0.01; ****, *P* ≤ 0.0001; ns, nonsignificant (*n* = 9 to 15). Data are represented as means, with error bars showing standard deviations. Download FIG S4, TIF file, 1.0 MB. Copyright © 2020 Singhal and Fang. 2020 Singhal and Fang. This content is distributed under the terms of the Creative Commons Attribution 4.0 International license.

### NO· directly inhibits PDF.

**FIG 5:**
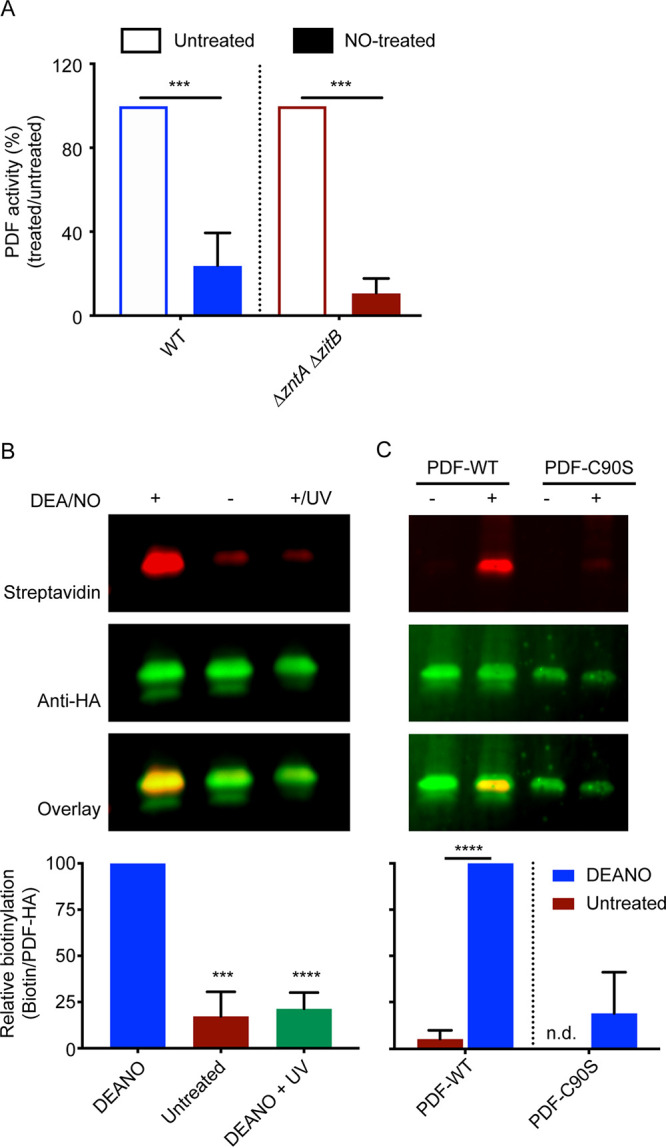
Nitric oxide directly modifies PDF. (A) *In vitro* treatment of whole-cell extracts with 200 μM DEA/NO inhibits PDF activity in both the WT and an isogenic Δ*zntA* Δ*zitB* mutant strain, suggesting a zinc-independent mechanism of NO·-mediated PDF inhibition. (B) Direct modification of PDF was confirmed by a biotin switch assay. A representative immunoprecipitation-Western blot (top) probed with a fluorescent streptavidin conjugate and anti-HA antibody to detect biotinylation and HA-tagged PDF, respectively, is shown. Densitometry of independent experiments (bottom) was performed to quantify the ratio of biotin to PDF. Significance was determined by an unpaired two-tailed *t* test. ***, *P* ≤ 0.001; ****, *P* ≤ 0.0001 (*n* = 4 [A] or 3 [B]). Data are represented as means, with error bars showing standard deviations. (C) The biotin switch assay was repeated with PDF-WT and PDF-C90S. The abrogation of Cys90 abolishes PDF nitrosylation by NO·. n.d., not detected.

To assess the possibility of direct PDF inhibition by NO·, PDF activity was measured in purified protein extracts of *S*. Typhimurium after treatment with the NO· donor SperNO. Extracts from both WT and Δ*zntA* Δ*zitB* mutant strains showed similar inhibition of PDF activity by NO· (Fig. 5A). As the NO· donor was added *ex vivo* after extract purification, this inhibition is concluded to result from the direct inhibition of PDF by NO· rather than by zinc mismetallation. It is interesting to note that NO· did not inhibit the activity of zinc-substituted PDF obtained from a zinc-treated Δ*zntA* Δ*zitB* strain (Fig. S5).

10.1128/mBio.01383-20.5 FIG S5 NO·-dependent inhibition of iron- or zinc-substituted PDF. Zinc-treated extracts from the WT or Δ*zntA* Δ*zitB* strain were treated with 200 μM DEA/NO for 10 min before measurement of PDF activity. Activity is plotted as a percentage of the total activity relative to that of respective NO·-untreated extracts. Native iron-bound PDF was found to be NO·-sensitive (blue bars), whereas zinc-bound protein was resistant (red bars). Significance was determined by an unpaired two-tailed *t* test. ***, *P* ≤ 0.001; ns, nonsignificant (*n* = 3). Data are represented as means, with error bars showing standard deviations. Download FIG S5, TIF file, 0.7 MB. Copyright © 2020 Singhal and Fang. 2020 Singhal and Fang. This content is distributed under the terms of the Creative Commons Attribution 4.0 International license.

The *S-*nitrosylation of PDF by NO· was confirmed by a biotin switch assay in which the *S*-nitrosyl group is replaced with biotin, and biotinylation is subsequently detected by immunoblotting. Whole-cell protein extracts of *Salmonella* expressing PDF-hemagglutinin (HA) on a multicopy inducible plasmid were treated with the NO· donor diethylamine NONOate (DEA/NO), and *S*-nitrosyl groups were exchanged with biotin. Biotinylated PDF protein was detected by fluorescence immunoblotting after the immunoprecipitation of HA-tagged PDF. This experiment showed the presence of biotinylated protein in extracts treated with the NO· donor but not in untreated extracts (Fig. 5B). The reversibility of *S-*nitrosylation was verified by the treatment of the nitrosylated protein with UV radiation.

As *S.* Typhimurium PDF contains two cysteine residues (Cys90 and Cys130), a biotin switch assay was performed using the PDF-C90S mutant in order to identify the cysteine residue modified by NO·. No significant biotinylation was observed after NO· treatment of extracts containing the PDF-C90S mutant protein (Fig. 5C), demonstrating that Cys90, but not Cys130, is a target of *S-*nitrosylation.

## DISCUSSION

Nitric oxide (NO·) exerts broad-spectrum antimicrobial activity by targeting protein thiols and metal centers required for diverse cellular processes. We have recently shown that NO· targeting of zinc metalloproteins mobilizes free intracellular zinc, which is ameliorated by the expression of zinc efflux transporters (8). The NO·-mediated mobilization of zinc from metalloproteins renders other proteins susceptible to zinc mismetallation, which has been suggested to be a mechanism of zinc toxicity (17, 18). For example, zinc can compete with manganese for binding to the solute-binding protein PsaA in Streptococcus pneumoniae, thereby inhibiting manganese accumulation (18). Zinc excess also results in mismetallation of the E. coli enzymes threonine dehydrogenase and ribulose-5-phosphate 3-epimerase, which contain mononuclear iron centers that are labile in the presence of oxidative stress (27). Of the transition metals, the stringent regulation of intracellular zinc concentrations is particularly important because zinc binds to ligands with high avidity but can diminish enzyme function when adventitiously bound (17). In this study, we show that NO· inactivates the essential mononuclear iron-containing enzyme PDF in *S.* Typhimurium through both zinc mismetallation and direct *S-*nitrosylation. This represents a novel mechanism of NO·-mediated antimicrobial activity.

PDF was initially believed to be a zinc metalloenzyme with low specific activity (28). Subsequently, it was recognized that the iron center of PDF is oxygen-labile (23), and PDF exhibits a higher avidity for zinc than for iron (19). Iron-bound PDF protein binds its formate substrate in a bidentate fashion, facilitating the activation of the bound carbonyl substrate by the metal serving as a Lewis acid. In contrast, the structure of zinc-bound PDF exhibits monodentate binding of formate; loss of activation by the metal ion can account for the reduction in enzyme activity (21). In the present study, we show that PDF overexpression ameliorates the inhibition of *Salmonella* growth by excess zinc (Fig. 1A and B). This suggests that PDF inhibition is an important contributor to zinc toxicity. PDF overexpression restores adequate enzyme activity, which would otherwise be inhibited by zinc overload, and relieves zinc toxicity. At higher zinc concentrations, PDF overexpression is no longer able to compensate for the inhibitory effects of zinc excess, which may indicate the existence of other targets of zinc overload. The residual activity of mismetallated PDF is resistant to oxidative damage, suggesting that zinc mismetallation can preserve residual PDF activity under oxidative stress conditions in which iron-bound PDF is unstable. Similar protective effects of manganese and cobalt have been suggested for E. coli PDF (24).

Both zinc and NO· are able to inhibit *Salmonella* growth in a dose-dependent manner. PDF overexpression suppresses *Salmonella* growth inhibition by zinc (Fig. 1), indicating that PDF mismetallation is a major consequence of zinc excess and contributes to impaired growth. *Salmonella* growth inhibition by NO· is enhanced in cells deficient in zinc efflux, consistent with the NO·-mediated mobilization of zinc, which would otherwise be exported from the cell to alleviate toxicity. However, PDF overexpression is unable to restore *Salmonella* growth in the presence of NO·, although it is able to abrogate the NO·-sensitive phenotype of a Δ*zntA* Δ*zitB* mutant strain. This is most likely due to the ability of NO· to inhibit growth by multiple mechanisms, including the inhibition of DNA replication and amino acid biosynthesis (4, 5), as well as the ability of NO· to inhibit PDF by both zinc-dependent and -independent mechanisms. In fact, PDF overexpression is detrimental to bacterial growth during nitrosative stress. The explanation for the paradoxical effects of PDF overexpression during nitrosative stress is unknown but might relate to the proportions of *S*-nitrosylated (inactive) and mismetallated (hypoactive) PDF at different NO· concentrations.

The essentiality of PDF and its conservation in all bacteria led to interest in developing novel antimicrobial agents that target PDF (29). Some rationally designed PDF inhibitors have even progressed into clinical trials (30). Our observations demonstrate that the endogenous antimicrobial mediator NO· also exploits the essentiality and conservation of PDF as a target to inhibit bacterial growth.

## MATERIALS AND METHODS

### Bacterial growth conditions.

Salmonella enterica serovar Typhimurium strain ATCC 14028s was used as the wild type for this study. *S.* Typhimurium was grown aerobically in Luria-Bertani (LB) medium at 37°C with shaking at 250 rpm. Ampicillin was used at 100 μg ml^−1^ as required. M9 minimal medium was used as a defined growth medium where mentioned.

### Strain and plasmid construction.

Strains and plasmids are listed in Table S1, and primers are listed in Table S2 in the supplemental material. E. coli strain DH10B was used as a host strain for cloning, and confirmed plasmids were subsequently electroporated into *S*. Typhimurium. The *def* gene was cloned into the EcoRI and KpnI sites of pTrc99A to generate pAS18 (p*def*) and into the BamHI and EcoRI sites of pGEX-2T to generate pAS19. A C-terminal HA tag was introduced in the coding region of *def* using oligonucleotides ASP62 and ASP35, and the amplified product was cloned into pTrc99A to generate pAS33. Site-specific mutagenesis of PDF-Cys90 was performed using HiFi assembly master mix (New England BioLabs) to generate pAS37 and pAS40. All plasmids were confirmed by DNA sequencing (Genewiz).

10.1128/mBio.01383-20.6 TABLE S1 Details of bacterial strains and plasmids used in this study. Download Table S1, DOCX file, 0.02 MB. Copyright © 2020 Singhal and Fang. 2020 Singhal and Fang. This content is distributed under the terms of the Creative Commons Attribution 4.0 International license.

10.1128/mBio.01383-20.7 TABLE S2 DNA sequences of the oligonucleotides used in this study. Download Table S2, DOCX file, 0.02 MB. Copyright © 2020 Singhal and Fang. 2020 Singhal and Fang. This content is distributed under the terms of the Creative Commons Attribution 4.0 International license.

### Zinc and nitric oxide sensitivity assays.

*S.* Typhimurium with either the pTrc99A vector or p*def* in WT strains (AS191 and AS192) or in strains lacking the ZntA and ZitB zinc efflux pumps (AS193 and AS194) was used. Strains AS351 and AS352 were used for growth assays to determine the effects of PDF-Cys90 and PDF-Cys130 mutations. The strains were grown overnight in 5 ml LB medium plus ampicillin and then diluted 1:1,000 into fresh LB medium plus ampicillin with 50 to 80 μM ZnSO_4_ or 0.6 to 2.5 mM spermine NONOate (SperNO) to a final volume of 300 μl in microtiter plate wells. To induce the expression of PDF, 0.5 mM isopropyl β-d-1-thiogalactopyranoside (IPTG) was included in the growth medium. Cultures were grown aerobically with shaking at 37°C in a Labsystems Bioscreen C machine (Growth Curves USA), and growth was monitored by measuring the OD_600_ every 15 min. Differences between cultures were determined by calculating the time to reach the 50% maximum OD_600_, and statistical significance was determined by an unpaired two-tailed *t* test.

### Whole-cell lysate preparation for enzyme assays.

Bacterial strains containing the p*def* plasmid (AS192 or AS194) or mutant p*def* derivatives (AS348 or AS349) were grown overnight in 5 ml LB medium and then diluted 1:100 in 50 ml fresh LB medium. Cells were induced for 1 h with 0.5 mM IPTG at an OD_600_ of 0.5 to 0.6. Cells were harvested, washed once with assay buffer (50 mM HEPES [pH 7.4] and 25 mM NaCl), and resuspended in 0.5 ml assay buffer containing 0.1 mM diethylenetriamine penta-acetic acid (DTPA). Cells were lysed mechanically using 0.1-mm silica beads, and cell debris was removed by centrifugation. The clear cell lysate was immediately used for enzyme assays. The protein content of cell lysates was estimated using the Coomassie protein assay reagent kit (Thermo Fisher Scientific). To measure the effects of zinc or NO· on enzyme activity, 125 μM ZnSO_4_ or 25 to 75 μM spermine NO· were added during the IPTG induction phase.

### Peptide deformylase enzyme assays.

Peptide deformylase activity was measured in assay buffer containing 5 mM NAD^+^, 1 U of formate dehydrogenase, and 1 mM formyl-Met-Ala-Ser peptide. A partially anaerobic environment was maintained using a glucose oxidase and catalase system by adding 20 mM d-glucose, 0.5 U of glucose oxidase, and 700 U of catalase (23). The reaction was started by adding freshly prepared cell lysates to the mixture. PDF removes the formyl group from formyl-Met-Ala-Ser to generate formate. Formate is then oxidized to CO_2_ and H_2_O by formate dehydrogenase, with the reduction of NAD^+^ to NADH, which is monitored by an increase in the OD_340_. The change in the absorbance per minute was converted to activity (micromoles per minute) units using a molar absorption coefficient of 6,220 M^−1^ cm^−1^, and the data were normalized to the protein amount.

To measure the effects of hydrogen peroxide (H_2_O_2_) or NO· on PDF activity *ex vivo*, lysates were prepared from zinc-treated AS192 or AS194, and equal amounts of the clarified lysates were incubated with 100 μM H_2_O_2_ or 200 μM DEA/NO for 10 min at room temperature (RT) before measuring the activity.

### Purification of GST-tagged PDF.

E. coli BL21(DE3) containing pAS19 (AS202) was grown overnight in 5 ml LB medium and diluted 1:100 in 25 ml fresh LB medium. Cells were induced with 1 mM IPTG for 1 h at an OD_600_ of 0.5 to 0.6. Harvested cells were resuspended in lysis buffer containing 50 mM Tris-HCl (pH 8.0), 150 mM NaCl, 1 mM dithiothreitol (DTT), 1 mM phenylmethylsulfonyl fluoride, and 1× protease inhibitor cocktail (Roche). Cells were lysed using sonication, and cell debris was removed by centrifugation. The cleared lysate was used to purify glutathione *S*-transferase (GST)–PDF using the Pierce GST spin purification kit according to the manufacturer’s directions. The protein quantity was estimated using the Coomassie protein assay reagent kit. The purified enzyme was aliquoted and stored at −80°C until further use.

### Preparation of metal-free apoPDF.

Freshly thawed GST-PDF (5.7 μM) was incubated with 25 mM EDTA in assay buffer at RT for 45 min to strip all metals from PDF to generate the apoenzyme. ApoPDF was used for assays at a 1:50 dilution in buffer containing 700 μM either Fe ammonium sulfate hexahydrate or Zn sulfate heptahydrate, sufficient to overcome the remaining EDTA.

### Detection of free zinc by FRET.

Freely available zinc was measured by analyzing fluorescence resonance energy transfer using flow cytometry essentially as described previously (8). *S.* Typhimurium wild-type or Δ*zntA* Δ*zitB* strains expressing ZapCV5 (AS168 and AS172) were grown overnight (∼20 h) and diluted in fresh LB medium to an OD_600_ of 0.6. Cells were treated with 0.5 mM spermine NO· and incubated at 37°C for 1 h with constant shaking. One milliliter of the culture was harvested by centrifugation, and cells were fixed with paraformaldehyde before analysis by flow cytometry (8). The experiment was repeated using M9 defined medium for untreated AS168 and AS172 strains.

### Biotin switch assay of PDF-HA.

The biotin switch method was adapted and modified from a previous study (31). AS265 or AS357 cultures expressing hemagglutinin (HA)-tagged derivatives of PDF or PDF-C90S grown overnight were diluted 1:100 in 200 ml LB medium and grown to an OD_600_ of 0.6. Cultures were induced with 1 mM IPTG for 1 h. Cells were pelleted, washed once with 50 ml cold phosphate-buffered saline (PBS), resuspended in 2.5 ml lysis buffer (250 mM HEPES [pH 7.7], 1 mM DTPA, and 0.1 mM neocuproine) containing 1% Triton X-100, and sonicated with a Microson ultrasonic cell disruptor XL instrument (Misonix). Cell debris was removed by centrifugation at 10,000 relative centrifugal force (rcf) for 15 min at 4°C. Low-molecular-weight thiols were removed with Econo-Pac 10DG desalting columns (BD Biosciences) according to the manufacturer’s recommendations. The total protein concentration was determined using the Coomassie protein assay reagent kit. The protein was aliquoted and stored at −80°C until further use. Two 3-ml samples (1 mg ml^−1^) were treated in dark conical tubes with 1 mM DEA/NO (Cayman Chemical) for 10 min at 37°C. A control sample was treated in parallel with 1 mM DEA (Sigma-Aldrich). As an additional negative control, one of the two DEA/NO-treated samples was exposed to UV light in the presence of 0.1 M mannitol and 5 mM *S*-methyl thiomethanesulfonate (MMTS) for 20 min to denitrosylate the proteins. To stop *S*-nitrosylation, samples were precipitated in 3 volumes of ice-cold 100% acetone and incubated for 20 min at −20°C. Samples were washed twice with 75% acetone and resuspended in 3 ml blocking buffer (lysis buffer with 2.5% SDS). All three samples were blocked with 40 mM MMTS for 60 min at 50°C with vortexing every 5 min. MMTS blocking was stopped by acetone precipitation followed by five washes with 75% acetone and final resuspension in 0.8 ml labeling buffer (lysis buffer with 1% SDS). To label *S*-nitrosylated proteins, samples were incubated for 60 min with 20 mM sodium l-ascorbate and 400 μM EZ-Link HPDP-biotin (Thermo Fisher Scientific). Labeling was performed on a rotator mixer at RT. To stop biotinylation, samples were acetone precipitated, followed by two washes with 75% acetone and final resuspension in 1 ml resuspension buffer (lysis buffer with 0.1% SDS). The protein concentration was determined, and 75 μg of biotin-labeled protein were immunoprecipitated with Pierce anti-HA magnetic beads (Thermo Fisher Scientific) according to the manufacturer’s recommendations. PDF-HA was eluted using SDS-PAGE sample buffer, subjected to SDS-PAGE, and transferred onto a polyvinylidene difluoride (PVDF) transfer membrane. Western blots were probed with Alexa Fluor 488 anti-hemagglutinin mouse monoclonal 16B12 antibody (Molecular Probes) and an Alexa Fluor 647 streptavidin conjugate (Molecular Probes). A FluorChem Q imaging system (Alpha Innotech) was used to visualize Western blots, and ImageJ version 1.51k software (Wayne Rasband, U.S. National Institutes of Health) was used for densitometry.
