# Supplementary figures and images for: Direct and Indirect Inhibition of Salmonella Peptide Deformylase by Nitric Oxide
Source: mBio. 2020 Nov 17;11(6):e01383-20. doi: 10.1128/mBio.01383-20 (PMC7683392; doi:10.1128/mBio.01383-20)

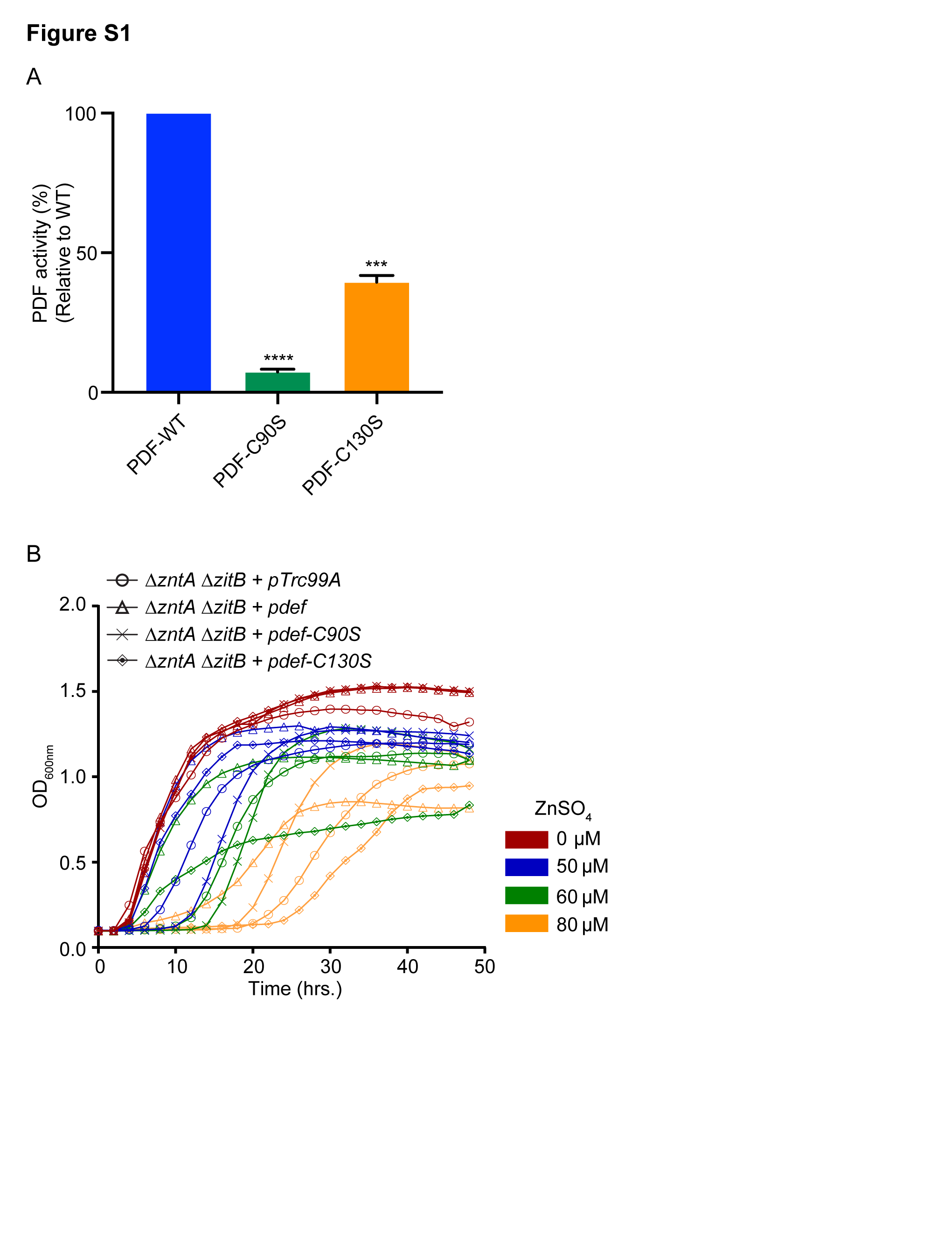

Supplement: FIG S1 [file mBio.01383-20-sf001.tif]

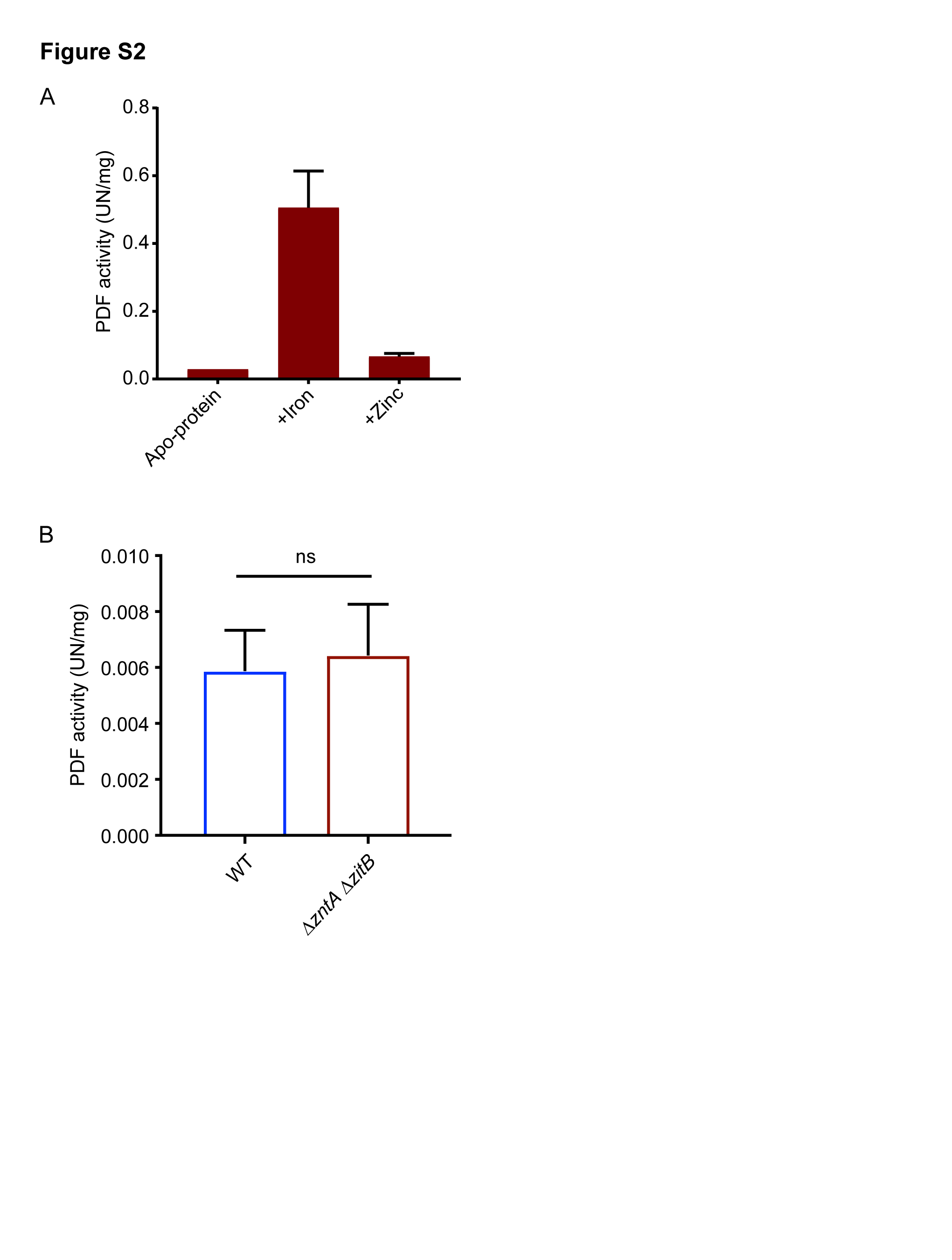

Supplement: FIG S2 [file mBio.01383-20-sf002.tif]

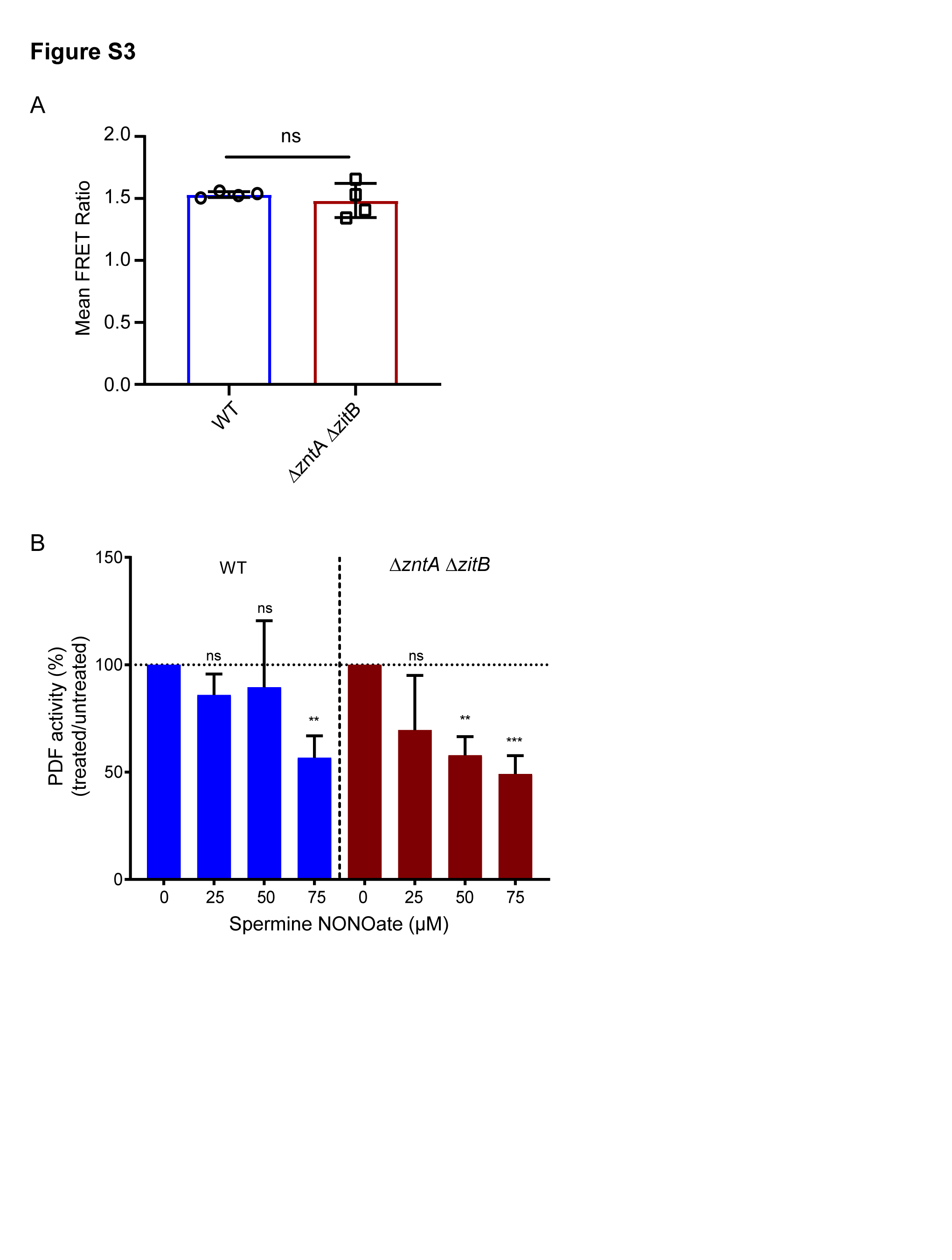

Supplement: FIG S3 [file mBio.01383-20-sf003.tif]

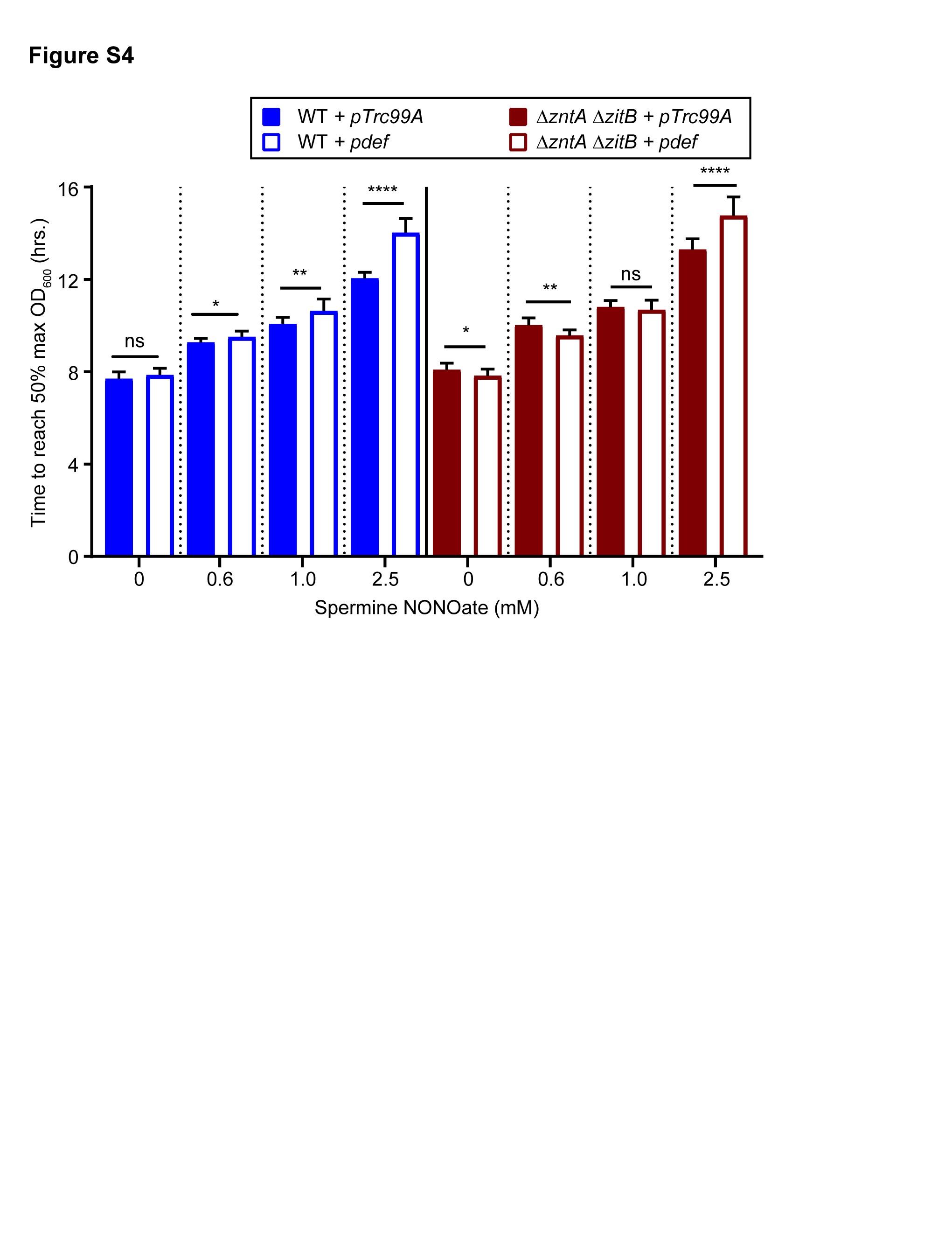

Supplement: FIG S4 [file mBio.01383-20-sf004.tif]

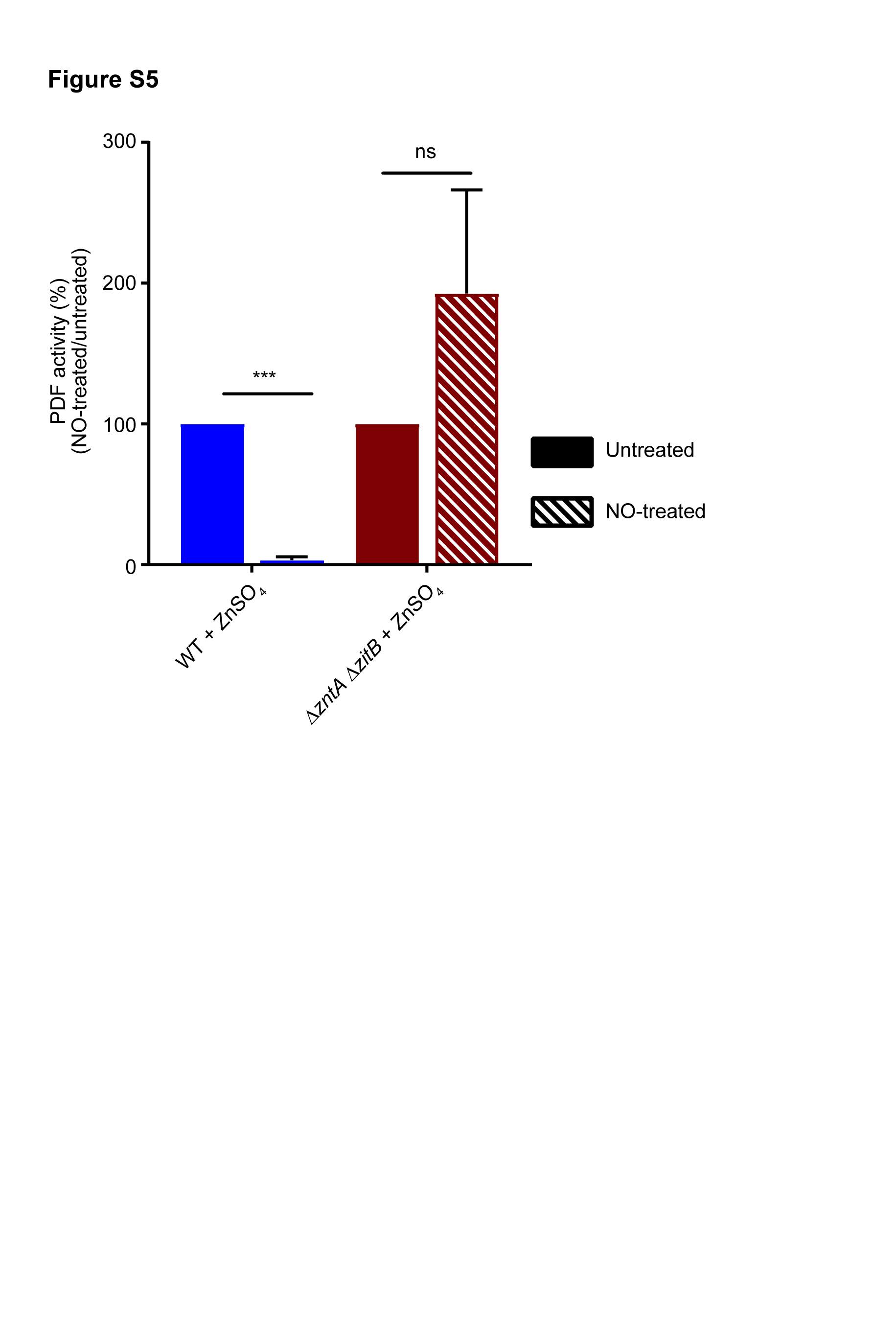

Supplement: FIG S5 [file mBio.01383-20-sf005.tif]
