# Supplementary material for: Direct and Indirect Inhibition of Salmonella Peptide Deformylase by Nitric Oxide
Source: mBio. 2020 Nov 17;11(6):e01383-20. doi: 10.1128/mBio.01383-20 (PMC7683392; doi:10.1128/mBio.01383-20)
Supplement: TABLE S1 [file mBio.01383-20-st001.docx]

**Supplementary Table S1. Strains and Plasmids**

| **Plasmid or Strain** | **Genotype** | **Source** |
| --- | --- | --- |
| pTrc99A | *bla* P_trc_ oricolE1 | Amann and Brosius 1985 |
| pGEX-2T | *bla* P_tac_-*gst* oricolE1 | Smith and Johnson 1988 |
| pAS18 | *bla* P_trc_-*def* oricolE1 | This study |
| pAS37 | *bla* P_trc_-*def-C90S* oricolE1 | This study |
| pAS38 | *bla* P_trc_-*def-C130S* oricolE1 | This study |
| pAS19 | *bla* P_tac_-*gst*-*def* oricolE1 | This study |
| pAS33 | *bla* P_trc_-*def-HA* oricolE1 | This study |
| pAS40 | *bla* P_trc_-*def-HA-C90S* oricolE1 | This study |
| BL21(DE3) | *E. coli* *B* F^–^ *dcm ompT hsdS_B_*(r_B_^-^ m _B_^-^) *gal* λ(DE3) | Novagen |
| JK237 | 14028s | ATCC |
| EF532 | Δ*zntA::FRT* Δ*zitB::FRT* | Frawley et al. 2018 |
| AS191 | 14028s/pTrc99A | This study |
| AS192 | 14028s/pAS18 | This study |
| AS193 | Δ*zntA::FRT* Δ*zitB::FRT* /pTrc99A | This study |
| AS194 | Δ*zntA::FRT* Δ*zitB::FRT* /pAS18 | This study |
| AS351 | Δ*zntA::FRT* Δ*zitB::FRT* /pAS37 | This study |
| AS352 | Δ*zntA::FRT* Δ*zitB::FRT* /pAS38 | This study |
| AS348 | 14028s/pAS37 | This study |
| AS349 | 14028s/pAS38 | This study |
| AS202 | BL21(DE3)/pAS19 | This study |
| AS265 | 14028s/pAS33 | This study |
| AS357 | 14028s/pAS40 | This study |

**References**

1. Amann E, Brosius J. 1985. "ATG vectors” for regulated high-level expression of cloned genes in *Escherichia coli*. Gene 40:183-90.
2. Smith DB, Johnson KS. 1988. Single-step purification of polypeptides expressed in *Escherichia coli* as fusions with glutathione S-transferase. Gene 67:31–40.
3. Frawley ER, Karlinsey JE, Singhal A, Libby SJ, Doulias PT, Ischiropoulos H, Fang FC. 2018. Nitric oxide disrupts zinc homeostasis in *Salmonella enterica* serovar Typhimurium. mBio 9:e01040-18
