# Supplementary material for: Direct and Indirect Inhibition of Salmonella Peptide Deformylase by Nitric Oxide
Source: mBio. 2020 Nov 17;11(6):e01383-20. doi: 10.1128/mBio.01383-20 (PMC7683392; doi:10.1128/mBio.01383-20)
Supplement: TABLE S2 [file mBio.01383-20-st002.docx]

**Supplementary Table S2. Oligonucleotides used in this study**

| **Primer** | **Sequence (5’-3’)** | **Use** |
| --- | --- | --- |
| ASP35 | CTGGAGAATTCATGTCAGTTTTGC | pAS18 and pAS33 construction |
| ASP36 | TTGGTACCTGTCCTTTAAGCCCGG | pAS18 construction |
| ASP37 | CTCTGGATCCATGTCAGTTTTGCAAG | pAS19 construction |
| ASP38 | GGTTCTGAATTCTTAAGCCCGGGCG | pAS19 construction |
| ASP91 | GAAGAAGGCAGTCTGTCGATTCCGGAACAG | pAS37 construction |
| ASP92 | GAATCGACAGACTGCCTTCTTCTATACCCG | pAS37 construction |
| ASP93 | CTGGCAATTAGCATTCAGCATGAGATGGATC | pAS38 construction |
| ASP94 | CATGCTGAATGCTAATTGCCAGCAAACCATC | pAS38 construction |
| ASP95 | GGAAACAGACCATGGAATTCATGTCAGTTTTGCAA | def mutagenesis |
| ASP96 | GATCCCCGGGTACCTGTCCTTTAAGCCCGGGCGTT | *def* mutagenesis |
| ASP62 | AAGGTACCTCAAGCGTAGTCCGGGACGTCGTACGGGTAAGCCCGGGCGTTCAGGCGGT | pAS33 construction |
